# Cathepsins and age-related macular degeneration: A Mendelian randomization study unveiling causal relationships

**DOI:** 10.1097/MD.0000000000042357

**Published:** 2025-05-09

**Authors:** Xiaoyan Han, Zhixiang Hua, Han Chen, Jin Yang

**Affiliations:** a Department of Ophthalmology and the Eye Institute, Eye and Ear, Nose, and Throat Hospital, Fudan University, Shanghai, PR China; b Key NHC Key Laboratory of Myopia (Fudan University), Laboratory of Myopia, Chinese Academy of Medical Sciences, Shanghai, PR China; c Shanghai Key Laboratory of Visual Impairment and Restoration, Shanghai, PR China.

**Keywords:** age-related macular degeneration, cathepsin, Mendelian randomization

## Abstract

Age-related macular degeneration (AMD) is a leading cause of vision impairment and blindness in older adults, profoundly affecting millions of individuals worldwide. Cathepsins are a crucial class of proteolytic enzymes that participates in multiple biological process. However, the role of cathepsins in AMD still remains unclear. This study aims to probe into the causal relationship between cathepsins and AMD using a 2-sample Mendelian randomization (MR). Instrumental variables associated with exposure (cathepsins) and the outcome (AMD) were sourced from published genome-wide association studies. To estimate the causal effects, methodologies such as inverse variance weighted, MR-Egger, and weighted median estimation (WM) were employed. Reverse MR and multivariate MR analyses were also performed. The elevated levels of cathepsin B significantly increased the risk of dry AMD, with an odds ratio (OR) of 1.068 (95% CI = 1.007–1.133) and a *P*-value of .029). Sensitivity analyses confirmed the robustness of these findings, with no evidence of heterogeneity or pleiotropy. Reverse MR analyses indicated that total AMD might elevate levels of cathepsin E (OR = 1.04, *P* = .029). Multivariate MR analysis showed significant associations between specific cathepsins and AMD subtypes, including cathepsin G and cathepsin O with significantly increasing risk. The study revealed a potential causal effect of cathepsin B on AMD, especially dry AMD. These findings provide potential therapeutic targets for AMD, and further research is needed to understand the underlying mechanisms.

## 1. Introduction

Age-related macular degeneration (AMD), a progressive chronic disease of the central retina, is a leading cause of vision impairment and blindness in older adults, profoundly affecting millions of individuals worldwide.^[1,2]^ The disease manifests primarily in 2 forms: wet AMD (wAMD) and dry AMD (dAMD), which are both associated with progressive visual impairment.^[3]^ Moreover, the pathophysiology of AMD is complex and multifactorial, involving genetic, age, environmental, and lifestyle risk factors.^[4]^ Notably, genetic polymorphisms play a significant role in AMD, with regulating complement, lipid, angiogenic, and extracellular matrix pathways being implicated.^[5]^ Nowadays, the therapeutic strategies of AMD mainly include anti-vascular endothelial growth factor (anti-VEGF) therapies for neovascular AMD, and laser therapies or surgical interventions for dAMD.^[4,6]^ Anti-VEGF drugs, such as ranibizumab and bevacizumab, can inhibit angiogenesis and have proven effective in slowing disease progression and improving visual outcomes.^[7]^ Additionally, recent phase 3 clinical trials with intravitreal anticomplement factors like pegcetacoplan and avacincaptad pegol have shown promise in reducing the progression of geographic atrophy, heralding potential new treatment avenues for dAMD.^[6]^ However, while these treatments have revolutionized the management of neovascular AMD, they come with potential systemic safety concerns that warrant further investigation. Therefore, future research needs to focus on elucidating the underlying mechanisms, exploring new treatment targets, and developing comprehensive, multi-faceted therapeutic approaches.

Cathepsins are a crucial class of proteolytic enzymes that belong to the cysteine, serine, and aspartic protease families. Structurally, cathepsins are classified into 3 major groups according to the residue in their catalytic triad: cysteine cathepsins (e.g., cathepsin B, L, and K), serine cathepsins (e.g., cathepsin A and G), and aspartic cathepsins (e.g., cathepsin D and E).^[8]^ During the process, cathepsins are synthesized as inactive proenzymes, or zymogens, and are required proteolytic cleavage for activation. The activation often occurs within the acidic environment of lysosomes, and is regulated at multiple levels, including gene expression, proteolytic activation, and inhibition by endogenous inhibitors such as cystatins and serpins.^[9–11]^ This precise regulation is critical, as dysregulation can lead to excessive or insufficient proteolytic activity, contributing to pathological conditions. Recent research has highlighted the versatile roles of cathepsins beyond their traditional lysosomal degradation functions, like apoptosis, antigen presentation, and extracellular matrix remodeling.^[12–16]^ This versatility underscores their involvement in various human diseases, including neurodegenerative disorders, cardiovascular diseases, cancer, and inflammatory conditions.^[13,17,18]^ It is reported that cathepsins, encompassing various subtypes such as cathepsin A, B, D, L, and Z, are present in the choroid.^[19]^ Moreover, these proteolytic enzymes are involved in the degradation of the extracellular matrix and protein metabolism, thereby playing a crucial role in preserving the normal structure and function of the choroid.^[20]^ Empirical studies have demonstrated that cathepsins B, Z, and L are instrumental in modulating the formation of choroidal neovascularization (CNV). Furthermore, the inhibition of these specific cathepsins has been shown to significantly reduce the extent of CNV lesions. In addition, the pathogenesis of AMD is related to oxidative stress, and the activity of cathepsins may be affected by oxidative stress, thereby participating in the pathological process of AMD.^[19–21]^ Furthermore, genetic mutations and polymorphisms in cathepsin genes have been linked to specific hereditary diseases, providing insights into their physiological relevance and potential as therapeutic targets.^[22–24]^ In recent years, the role of cathepsins in ocular diseases has emerged as a crucial area of study, and the relationship between cathepsins and AMD still needs further investigated.

Mendelian randomization (MR) analysis is a statistical method that exploits the inherent properties of common genetic variations to evaluate the causal association between environmental exposures and diseases, by mimicking a lifelong randomized controlled trial.^[25,26]^ Of these, 2-sample MR analysis can utilize single-nucleotide polymorphism (SNP)-exposure and SNP-outcome associations from independent GWASs and combine them into a single causal estimate.^[27]^ In our ongoing study, genetic variants associated with the exposure (in this study, cathepsins) are identified as proxies instrumental variables (IVs) and are then tested for their combined effect on the outcome (in this study, AMD). Thus, we performed a 2-sample MR analysis to probe into the potential relationship between the cathepsins and AMD, utilizing data obtained from whole-genome association studies.

## 2. Materials and methods

### 2.1. Study design

**Figure 1.**
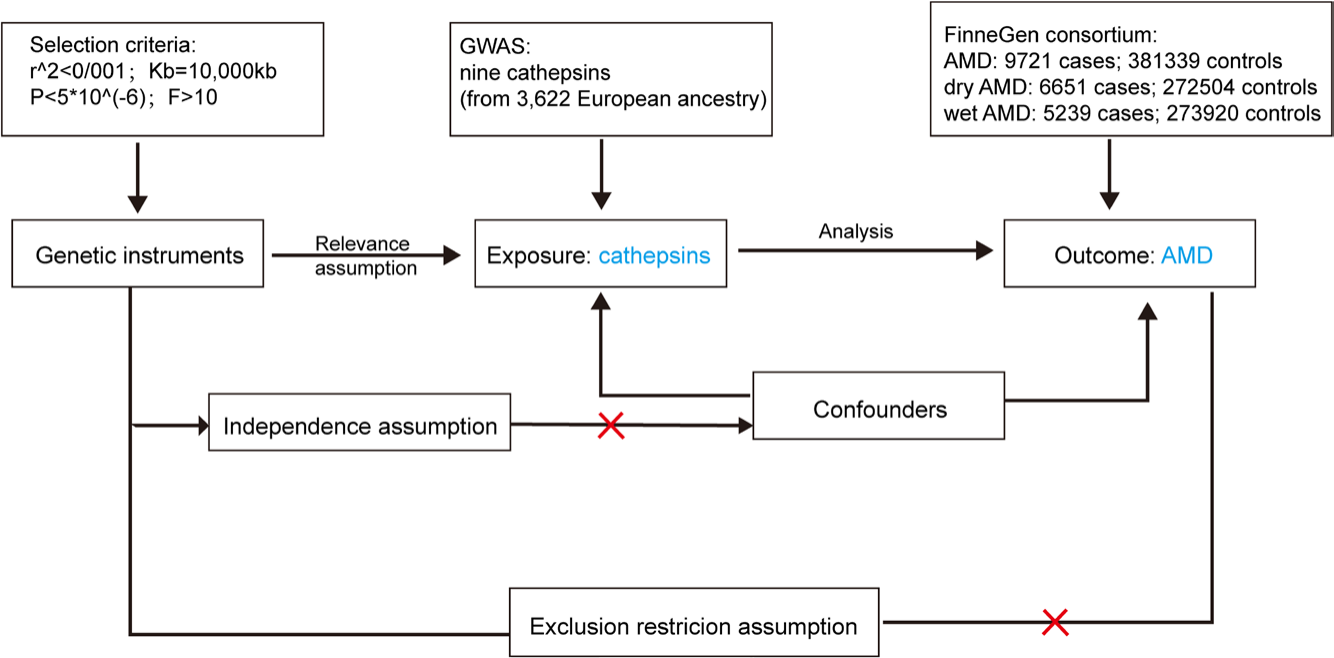
Flowchart of the study design.

In this study, two-sample MR (TSMR) were employed to evaluate the causal relationship between 9 cathepsins and the risk of developing AMD. Genetic variants associated with exposure (in this study, cathepsins) are identified as proxies (IVs) and are then tested for their combined effect on the the outcome (in this study, AMD). The MR analysis was guided by 3 primary hypotheses: strong association hypothesis: selected SNPs must exhibit a strong association with levels of cathepsins (exposure). No confounding hypothesis: SNPs should not be associated with any potential confounding factors that could influence both cathepsin levels and AMD risk. Exclusive influence hypothesis: SNPs should influence the risk of AMD exclusively through their impact on cathepsin levels, indicating the absence of genetic pleiotropy. The flow chart of this study is shown in Figure 1. All participants in the studies provided written informed consent, and the research protocols were approved by the respective institutional ethics committees. Genetic samples were sourced from separate genome-wide association studies (GWAS) databases to minimize overlap and biases. This thorough approval process eliminated the need for additional ethical clearances for this MR study.

### 2.2. GWAS for instrumental variables and outcomes

The MR analysis utilized genetic instruments to measure the levels of 9 cathepsin sourced from the INTERVAL study involving 3301 participants from European descent.^[28]^ Public access to the data is available through the GWAS database website (https://gwas.mrcieu.ac.uk.).

The genetic data for AMD were sourced from the latest FinnGen consortium database (DF10).^[29]^ This extensive database comprises 9721 cases of AMD, accompanied by 381,339 control individuals, ensuring a robust dataset for thorough analysis. Within this dataset, the dAMD subset encompasses 6651 cases and is complemented by 272,504 controls. Additionally, the wAMD data consist of 5239 cases and 273,920 controls. Together, these comprehensive datasets provide a valuable resource for investigating the genetic factors associated with different forms of AMD in a large population. Further specifics and data details are available via the official FinnGen website (https://www.finngen.fi/en/).

### 2.3. Selection of instrumental variables

The selection of SNPs as instrumental variables was essential for establishing the accuracy of the causal relationship between cathepsins and AMD. To affirm the validity and accuracy of this relationship, rigorous steps were undertaken to identify and select the most suitable SNPs associated with the exposure factors. Given the limited pool of SNPs available for MR analysis, a significance threshold was set at *P* < 5 × 10^−6^ to identify those SNPs that demonstrated strong associations with the exposures being studied. Additionally, to prevent the influence of linkage disequilibrium, an *r*^2^ threshold of 0.001 and a clump window size of 10,000 kilobases were applied. Moreover, it was imperative to confirm that the selected SNPs were not associated with any confounding factors that might distort the observed relationships between the exposures and outcomes. Consequently, each SNP was rigorously verified to ensure that it affected the outcome exclusively through its impact on the exposure factors, thereby maintaining a clear and unambiguous path of causality within the analysis.

### 2.4. Statistical methods

To test the hypothesis that genetic instruments affect AMD by influencing cathepsins, we utilized MR analysis methods. Then, we performed inverse variance weighted (IVW) analysis of MR estimates for all instrument variables to derive the overall MR estimate for the effect of cathepsin on the AMD. The IVW method was primarily utilized to assess causality, yielding significant results when the *P*-value fell below .05. The causality was evaluated using the odds ratio (OR) and a 95% confidence interval (CI), with an OR value <1 indicating a protective effect and an OR value >1 signifying a risk factor.

To test the hypothesis that genetic instruments affect AMD by influencing cathepsins, we utilized MR analysis methods. Specifically, the IVW approach was applied to derive the overall MR estimate for the effect of cathepsins on AMD across all instrumental variables. The IVW method, which assumes all genetic instruments are valid and free of horizontal pleiotropy, was selected for its statistical power and efficiency in combining SNP-level effects. The IVW method primarily assessed causality, with significance determined at a threshold of *P* < .05. Causality was evaluated using ORs and 95% CI, where an OR <1 indicated a protective effect and an OR >1 suggested a risk factor.

To affirm the robustness of the results, the MR-Egger and Weighted Median methods served as supplementary analyses. These methods provide alternative perspectives on causality and help mitigate potential biases from pleiotropy or invalid instruments. Additionally, a reverse MR analysis was conducted to investigate the possibility of reverse causation, examining whether AMD could influence cathepsin levels. In the multivariable MR analysis, we assessed the direct causal effects of each cathepsin on different AMD subtypes, considering multiple exposures simultaneously. The criteria for selecting specific cathepsins as adjustment variables were based on their known biological relevance and the need to account for pleiotropic effects that might confound the analysis. This approach enhances the specificity of causal estimates by isolating the effect of individual cathepsins while adjusting for potential shared genetic influences. In this study, the threshold of *P* < .05 was used to determine statistical significance.

### 2.5. Sensitivity analysis

The efficacy of the MR approach hinges on a pivotal assumption of pleiotropy absence. Consequently, it is imperative to assess the likelihood of pleiotropy when employing MR techniques. In order to delve deeper into the robustness of our findings against potential pleiotropic effects, we conducted a series of sensitivity analyses. First, heterogeneity was estimated using Cochran *Q* test. Cochran *Q* is a measure of heterogeneity among causal estimates and serves as an indicator of the presence of horizontal pleiotropy.^[30]^ Second, we used the weighted median and MR-Egger methods as mentioned above.^[31,32]^ Specifically, a weighted median is robust to invalid instruments and can provide consistent estimation even when up to 50% of the weight is from invalid SNPs. The MR-Egger method may provide correct estimates as long as instrument strength is independent of the direct effect assumption is satisfied. The *P*-value of the MR-Egger intercept indicates the presence of horizontal heterogeneity, with a *P*-value < .05 indicating the possibility of heterogeneity. Finally, Leave-one-out analysis was conducted to exclude SNPs with extreme influences.

### 2.6. Software and tools

Conduct all statistical analyses using the R software (4.3.3), utilizing specialized packages such as TSMR and MR-PRESSO for MR analyses. R language packages, such as ggplot2 and forest plot package, were applied to plot scatter plots and forest plots.

## 3. Results

### 3.1. Association of various cathepsins with different subtypes of AMD

This study employed 2-Sample MR analyses to investigate the effects of 9 cathepsins (B, E, F, G, H, L2, O, S, and Z) on the overall risk and various histological subtypes of AMD, including total AMD, dAMD, and wAMD. Notably, the univariable MR analysis indicated that elevated levels of cathepsin B significantly increased the risk of dry AMD, with an OR of 1.068 (95% CI = 1.007–1.133) and a *P*-value of .029 (Table 1). However, the IVW method did not reveal any causal associations between the other types of cathepsins and overall AMD or its major histological subtypes.

**Table 1:** Effect of cathepsins on AMD and its histological subtypes estimated by univariable Mendelian randomization analysis.

Cathepsin	SNPs	Inverse variance weighted		MR-Egger		Weighted median	
		OR (95% CI)	*P*-value	OR (95% CI)	*P*-value	OR (95% CI)	*P*-value
Cathepsin B							
AMD	20	1.051 (0.985–1.122)	.131	1.033 (0.882–1.209)	.695	1.009 (0.935–1.088)	.820
Dry AMD	20	1.068 (1.007–1.133)	.029*	1.038 (0.902–1.194)	.612	1.007 (0.924–1.097)	.874
Wet AMD	20	1.051 (0.957–1.155)	.299	1.061 (0.844–1.335)	.619	1.041 (0.940–1.153)	.436
Cathepsin E							
AMD	9	0.974 (0.897–1.056)	.521	.941 (0.816–1..084)	.427	.936 (0.855–1.024)	.151
Dry AMD	9	0.955 (0.890–1.025)	.204	0.971 (0.863–1.093)	.638	0.948 (0.856–1.051)	.311
Wet AMD	9	0.988 (0.866–1.127)	.854	0.906 (0.726–1.131)	.413	0.939 (0.842–1.047)	.253
Cathepsin F							
AMD	12	1.029 (0.963–1.100)	.399	1.007 (0.856–1.185)	.933	1.021 (0.940–1.109)	.620
Dry AMD	12	1.0233 (0.958–1.093)	.495	0.963 (0.826–1.124)	.645	1.015 (0.929–1.108)	.747
Wet AMD	12	1.017 (0.928–1.114)	.720	1.090 (0.876–1.356)	.457	0.951 (0.856–1.057)	.354
Cathepsin G							
AMD	12	1.028 (0.959–1.102)	.435	1.124 (0.971–1.300)	.148	1.055 (0.964–1.155)	.245
Dry AMD	12	0.980 (0.903–1.065)	.639	1.057 (0.882–1.266)	.564	0.966 (0.866–1.078)	.538
Wet AMD	12	1.099 (0.972–1.243)	.132	1.251 (0.960–1.630)	.129	1.098 (0.965–1.249)	.158
Cathepsin H							
AMD	11	1.001 (0.966–1.036)	.976	0.965 (0.920–1.013)	.185	0.988 (0.951–1.027)	.544
Dry AMD	11	1.008 (0.959–1.059)	.764	0.976 (0.914–1.043)	.491	0.992 (0.948–1.037)	.719
Wet AMD	11	0.978 (0.933–1.026)	.362	0.927 (0.869–0.988)	.004	0.957 (0.911–1.006)	.081
Cathepsin O							
AMD	12	0.968 (0.902–1.039)	.369	1.010 (0.857–1.191)	.904	0.935 (0.844–1.036)	.198
Dry AMD	12	0.936 (0.861–1.018)	.123	1.004 (0.828–1.218)	.967	0.912 (0.815–1.021)	.109
Wet AMD	12	1.009 (0.918–1.108)	.860	1.024 (0.824–1.272)	.835	0.972 (0.855–1.105)	.661
Cathepsin S							
AMD	23	0.980 (0.927–1.037)	.484	0.885 (0.816–0.960)	.008	0.945 (0.886–1.007)	.080
Dry AMD	23	0.981 (0.917–1.050)	.579	0.877 (0.791–0.971)	.020	0.926 (0.858–0.999)	.046
Wet AMD	23	0.984 (0.928–1.044)	.593	0.914 (0.826–1.010)	.093	0.909 (0.840–0.984)	.018
Cathepsin L2							
AMD	12	0.928 (0.858–1.004)	.063	0.967 (0.788–1.186)	.754	0.926 (0.835–1.028)	.150
Dry AMD	12	0.921 (0.837–1.015)	.096	0.742 (0.583–0.944)	.036	0.975 (0.852–1.116)	.713
Wet AMD	12	0.909 (0.807–1.024)	.118	1.245 (0.950–1.631)	.143	0.903 (0.781–1.043)	.165
Cathepsin Z							
AMD	13	1.004 (0.953–1.057)	.879	0.954 (0.884–1.031)	.260	0.962 (0.900–1.029)	.249
Dry AMD	13	1.016 (0.950–1.087)	.642	0.933 (0.852–1.022)	.165	0.973 (0.886–1.056)	.510
Wet AMD	13	0.973 (0.912–1.039)	.417	0.954 (0.861–1.057)	.392	0.957 (0.883–1.037)	.286

AMD = age-related macular degeneration, CI = confidence interval, MR = Mendelian randomization, OR = odds ratio.

*Indicates *P* value < .05.

### 3.2. MR sensitivity analysis

**Figure 2.**
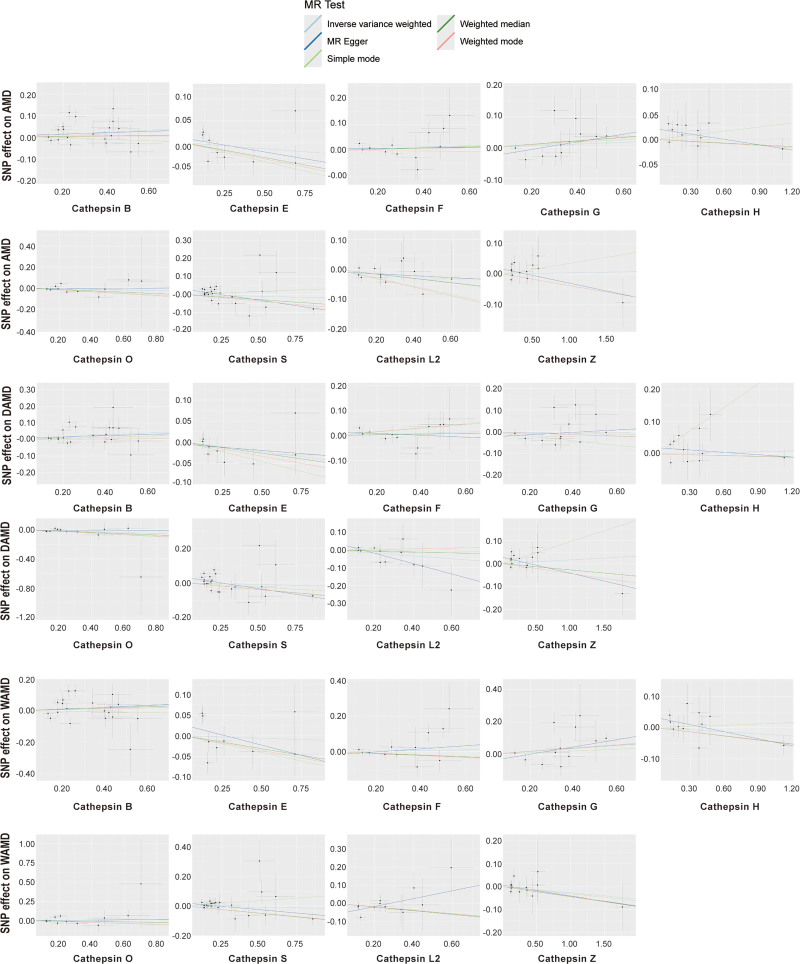
Scatter plots of MR analysis for cathepsins and AMD subtypes. Each point represents the effect of a SNP on both the exposure (cathepsins) and the outcome (AMD subtypes). Different colored regression lines indicate causal effects estimated using various algorithms. AMD = Age-related macular degeneration, MR = Mendelian randomization SNP = single-nucleotide polymorphism.

To address potential overlooks of pleiotropic effects by the IVW method, sensitivity analyses were conducted. Cochran *Q* test indicated no significant heterogeneity among the SNPs (*P* > .05). Furthermore, both the weighted media and MR-Egger intercept showed no evidence of horizontal pleiotropy. The scatter plots of these analyses, as illustrated in Figure 2, highlighting the consistency of the findings across different MR methods. Lastly, the results of the leave-one-out analysis further demonstrated the robustness of these findings.

### 3.3. Reverse MR analysis

To explore the potential, reverse causality, we conducted reverse MR analyses. The results revealed no reverse causation between cathepsin B and all kinds of AMD risks (Table 2). Interestingly, the analysis suggested that total AMD might elevate levels of cathepsin E (Table 2). Cathepsin E, an intracellular aspartic protease, is integral to various physiological and pathological processes, including intracellular protein metabolism, antigen processing, immune responses, tumorigenesis and development, and neurological disorders.^[33–35]^ Despite its established roles, there is a lack of research investigating the involvement of cathepsin E in the pathogenesis of AMD. Drawing on its mechanisms of action in other diseases, we hypothesize that cathepsin E may contribute to the onset and progression of AMD through the regulation of mitochondrial function. However, the role of cathepsin E in the pathogenesis of AMD needs further study. In addition, the IVW method supported this observation with statistically significant OR (OR = 1.040, 95% CI = 1.004–1.078, *P* = .029), and no pleiotropy was indicated in the tests.

**Table 2:** Effect of AMD and its histological subtypes on cathepsins estimated by reverse Mendelian randomization analysis (ranked by *P*-value).

Exposure	Outcome	Methods	Number of SNPs	*P*-value	OR	OR 95% LCI	OR 95% UPCI
AMD	Cathepsin E	IVW	164	.029*	1.040	1.004	1.078
	Cathepsin H	IVW	164	.193	1.024	0.988	1.061
	Cathepsin F	IVW	164	.296	1.020	0.983	1.059
	Cathepsin B	IVW	164	.406	0.985	0.950	1.021
	Cathepsin L2	IVW	164	.500	0.987	0.950	1.025
	Cathepsin O	IVW	164	.503	1.012	0.977	1.049
	Cathepsin Z	IVW	164	.547	1.011	0.975	1.050
	Cathepsin G	IVW	164	.732	0.993	0.957	1.032
	Cathepsin S	IVW	164	.923	0.998	0.964	1.034
dAMD	Cathepsin E	IVW	127	.163	1.025	0.990	1.062
	Cathepsin F	IVW	127	.184	1.025	0.988	1.064
	Cathepsin Z	IVW	127	.208	1.022	0.988	1.057
	Cathepsin G	IVW	127	.255	0.980	0.947	1.015
	Cathepsin L2	IVW	127	.296	0.982	0.949	1.016
	Cathepsin O	IVW	127	.406	1.015	0.981	1.050
	Cathepsin S	IVW	127	.727	0.994	0.959	1.030
	Cathepsin H	IVW	127	.797	0.996	0.962	1.030
	Cathepsin B	IVW	127	.848	0.997	0.963	1.031
wAMD	Cathepsin H	IVW	140	.052	1.035	1.000	1.072
	Cathepsin E	IVW	140	.055	1.031	0.999	1.064
	Cathepsin O	IVW	140	.160	1.023	0.991	1.056
	Cathepsin B	IVW	140	.254	1.020	0.986	1.054
	Cathepsin G	IVW	140	.267	0.982	0.952	1.014
	Cathepsin F	IVW	140	.275	1.019	0.985	1.053
	Cathepsin S	IVW	140	.296	1.017	0.985	1.050
	Cathepsin Z	IVW	140	.384	1.016	0.980	1.053
	Cathepsin L2	IVW	140	.569	0.991	0.960	1.023

AMD = age-related macular degeneration, CI = confidence interval, dAMD = dry AMD, IVW = inverse variance weighted, LCI = low confidence interval, MR = Mendelian randomization, OR = odds ratios, SNPs = single-nucleotide polymorphisms, UPCI = UP confidence interval, wAMD = wet AMD.

*Indicates *P* value < .05.

### 3.4. Multivariable MR analysis

**Figure 3.**
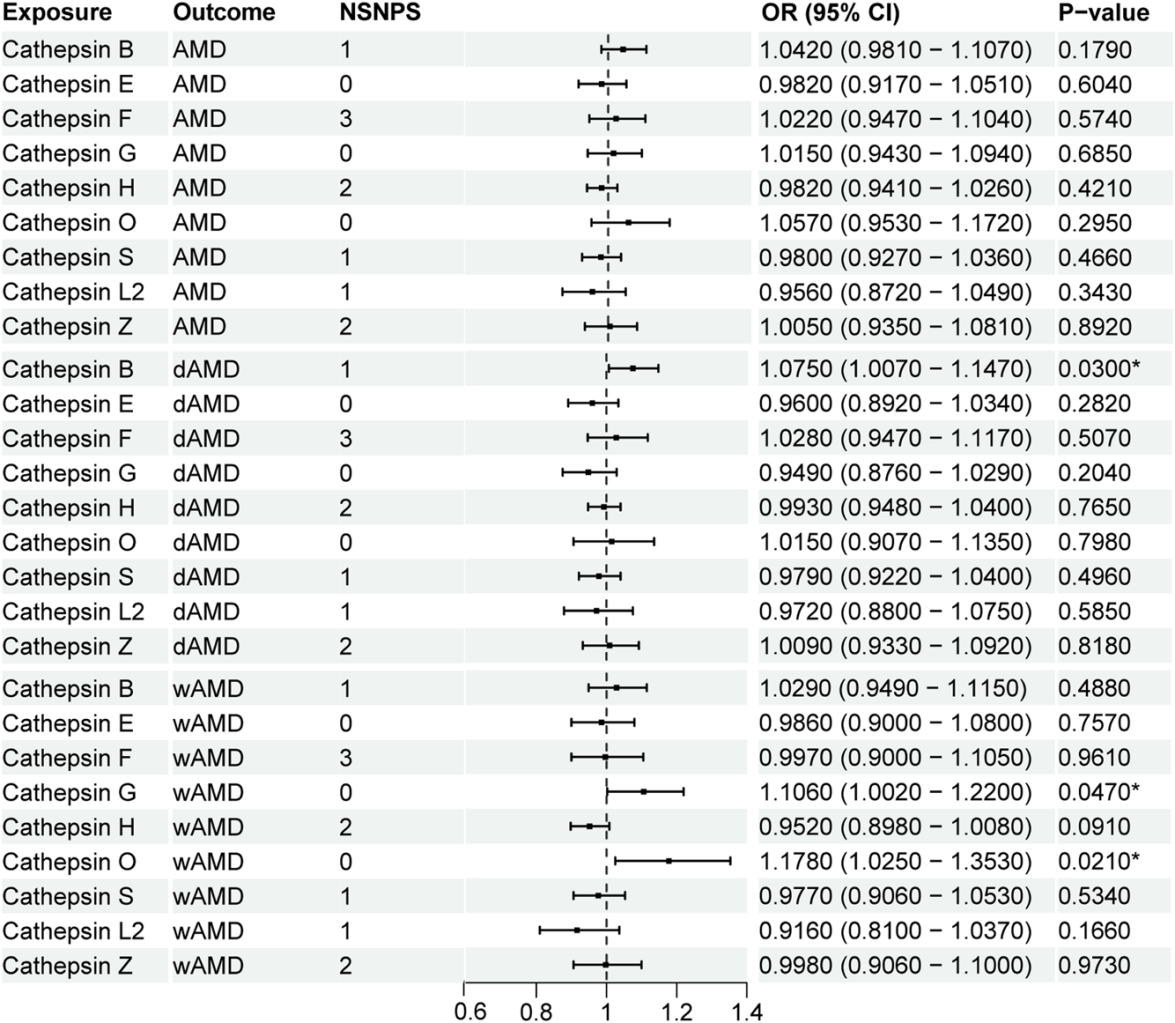
Forest plot of OR in multivariate MR analysis for the association between cathepsins and AMD. Linear forest plot illustrating the OR from the multivariate MR analysis for the association between cathepsins and AMD. Each horizontal line represents an OR with its corresponding confidence interval, providing a clear visualization of effect sizes across different models or variables. Asterisks (*) indicate statistically significant results with *P*-values < .05. AMD = age-related macular degeneration, MR = Mendelian randomization, OR = odds ratio.

We conducted a multivariable MR analysis to assess the genetic predisposition involving multiple cathepsins in relation to the risk of different AMD subtypes. However, after adjusting for the influence of other cathepsins, no statistically significant causal associations were observed between multiple cathepsins and total AMD (Table 3). However, the analysis revealed that elevated levels of cathepsin B were robustly associated with an increased risk of dAMD after adjustment for the effects of other cathepsins. This association was quantified with an OR of 1.075 (95% CI = 1.007–1.147) and a *P*-value of .030 (Table 3). Additionally, in wAMD, cathepsin G and cathepsin O were both found to significantly increase risk. To be specific, cathepsin G and cathepsin O were both found to significantly increase risk of in wAMD by multiple MR analysis. Cathepsin G is a serine protease primarily produced by neutrophils and can play a role in inflammation related diseases by participating in immune responses. Thus, cathepsin G may participate in the occurrence and development of wAMD by regulating the production of cytokines and chemokines, activating and shedding cell surface receptors, and promoting apoptosis.^[36,37]^ For cathepsin O, it is a cysteine protease capable of catalyzing the cleavage of peptide bonds, thereby contributing to protein degradation. In wAMD, it may play a role in the pathogenesis and progression of the disease by degrading the extracellular matrix and facilitating angiogenesis.^[19,20]^ Cathepsin G was associated with an increased risk of wAMD (OR = 1.106, 95% CI = 1.002–1.220, *P* = .047), and cathepsin O was even more strongly linked to increased risk (OR = 1.178, 95% CI = 1.025–1.353, *P* = .021), as depicted in Figure 3.

**Table 3:** Forest plot of multivariate MR analysis results (ranked by *P*-value).

Exposure	Number of SNPs	*P*-value (IVW)	OR	OR 95% LCI	OR 95% UPCI
AMD					
Cathepsin B	1	.179	1.042	0.981	1.107
Cathepsin O	0	.295	1.057	0.953	1.172
Cathepsin L2	1	.343	0.956	0.872	1.049
Cathepsin H	2	.421	0.982	0.941	1.026
Cathepsin S	1	.466	0.980	0.927	1.036
Cathepsin F	3	.574	1.022	0.947	1.104
Cathepsin E	0	.604	0.982	0.917	1.051
Cathepsin G	0	.685	1.015	0.943	1.094
Cathepsin Z	2	.892	1.005	0.935	1.081
**dAMD**					
Cathepsin B	1	.030*	1.075	1.007	1.147
Cathepsin G	0	.204	0.949	0.876	1.029
Cathepsin E	0	.282	0.960	0.892	1.034
Cathepsin S	1	.496	0.979	0.922	1.040
Cathepsin F	3	.507	1.028	0.947	1.117
Cathepsin L2	1	.585	0.972	0.880	1.075
Cathepsin H	2	.765	0.993	0.948	1.040
Cathepsin O	0	.798	1.015	0.907	1.135
Cathepsin Z	2	.818	1.009	0.933	1.092
**wAMD**					
Cathepsin Z	2	.973	0.998	0.906	1.100
Cathepsin O	0	.021*	1.178	1.025	1.353
Cathepsin G	0	.047*	1.106	1.002	1.220
Cathepsin H	2	.091	0.952	0.898	1.008
Cathepsin L2	1	.166	0.916	0.810	1.037
Cathepsin B	1	.488	1.029	0.949	1.115
Cathepsin S	1	.534	0.977	0.906	1.053
Cathepsin E	0	.757	0.986	0.900	1.080
Cathepsin F	3	.961	0.997	0.900	1.105

AMD = age-related macular degeneration, CI = confidence interval, dAMD = dry AMD, IVW = inverse variance weighted, LCI = low confidence interval, MR = Mendelian randomization, OR = odds ratio, SNPs = single-nucleotide polymorphisms, UPCI = UP confidence interval, wAMD = wet AMD.

*Indicates *P* value < .05.

## 4. Discussion

Age-related macular degeneration is a progressive, vision-threatening disease that affects the elderly population. The development and progression of AMD involves a highly complex factors in which the genetic variation influences the disease risk substantially.^[2]^ In this study, we analyzed the causal link between 9 different cathepsins and the risk of various histological subtypes of AMD using MR analyses. Based on the analysis of univariable MR, reverse MR, and multivariable MR, our findings indicate that cathepsin B is a significant risk factor for AMD, particularly for the dAMD. Furthermore, no evidence of reverse causality for cathepsin B was observed.

AMD can be categorized into early and advanced stages clinically. The advanced stage poses a significant threat to visual acuity and encompasses both dAMD and wAMD. dAMD is characterized by geographic atrophy of the retinal pigment epithelial (RPE), photoreceptors, and choriocapillaris, leading to progressive retinal cell loss and a subsequent decline in visual acuity. In contrast, wAMD is marked by choroidal neovascularization (CNV), which results in subretinal leakage of blood, lipids, and fluids, as well as the formation of fibrous scars.^[3]^ Although the pathogenesis of AMD remain unclear, chronic inflammation, lipid deposition, oxidative stress, and impaired maintenance of the extracellular matrix are closely linked to the pathogenesis of AMD.^[38,39]^ Cathepsins are a group of proteolytic enzymes involved in various physiological processes, including protein degradation, antigen presentation, and tissue remodeling.^[16]^ Research has reported that cathepsin can be involved in the pathogenesis of AMD in multiple ways, such as the activity of cathepsin in Bruch membrane and RPE cells affecting extracellular matrix remodeling, which in turn affects the progression of AMD. Meanwhile, Cathepsin can participate in the expression and activity of VEGF, promoting the formation of new blood vessels in wet AMD.^[40,41]^ In recent years, the role of cathepsins in ocular diseases has emerged as a crucial area of study, given their involvement in maintaining ocular homeostasis and their contribution to the pathogenesis of several eye conditions.

In the present study, we identified cathepsin B as a significant risk factor for AMD, with a particular emphasis on its association with dAMD. The findings derived from the IVW methods were corroborated by other complementary analytical approaches, and there was no indication of pleiotropy or reverse causality. In contrast, we found no significant association between cathepsin B and wAMD. Cathepsin B is an important lysosomal cysteine protease that plays a key role in many physiological and pathological processes. Its activity is closely related to the occurrence and progression of many diseases, including cancer, neurodegenerative diseases, cardiovascular diseases, liver diseases and parasitic infections. Previous studies have explored possible mechanisms related to cathepsin B and tumors, suggesting that it can promote tumor cell invasion and metastasis by degrading extracellular matrix and basement membrane, and can activate matrix metalloproteinases (MMPs) and VEGF to promote tumor angiogenesis, thus participating in the occurrence and development of various cancers (such as breast cancer, colorectal cancer, prostate cancer, and brain tumor).^[16,42]^ Besides, studies indicated that Cathepsin B can degrade extracellular matrix, weaken the stability of arterial wall, promote plaque rupture, and participate in atherosclerosis.^[43]^ Furthermore, the relationship between cathepsin B and glaucoma had been investigated, suggesting that cathepsin B was significantly overexpressed in the trabecular meshwork of glaucoma patients. These enzymes contribute to the remodeling of the extracellular matrix, which is crucial for the maintenance of intraocular pressure. Dysregulated cathepsin activity may lead to impaired outflow of aqueous humor, subsequently causing elevated intraocular pressure and optic nerve damage.^[44,45]^ Research has demonstrated that in AMD, the aberrant activity of cathepsin B can disrupt the equilibrium in the degradation of extracellular matrix (ECM) components. Specifically, an upregulation in cathepsin B activity may result in excessive degradation of specific ECM components, thereby compromising the structural integrity of the retina. This disruption can adversely affect the exchange of materials and interactions between RPE cells and the choroid, ultimately leading to RPE cell dysfunction and facilitating the progression of AMD. Moreover, cathepsin B can participate in the degradation process of reactive oxygen species (ROS) within RPE cells. Its abnormality may cause the accumulation of incompletely degraded ROS components within RPE cells, affect the normal metabolism of the cells, and then damage the function of RPE cells. In addition, in the pathological progression of dAMD, cathepsin B may impair mitochondrial function, leading to the overproduction of ROS. These ROS can subsequently damage cellular biomacromolecules, resulting in oxidative stress that contributes to cellular aging and apoptosis in RPE cells, thereby advancing the progression of AMD.^[17,19,41,44,45]^ However, the mechanism of cathepsin B in AMD is still complex and unclear, and further research is needed to clarify its role.

Furthermore, the results of reverse MR analyses indicated that total AMD might elevate levels of cathepsin E. Cathepsin E is an endopeptidase belonging to the aspartic protease family, which plays a key role in many physiological functions such as protein degradation, immune response regulation and apoptosis. It has been reported that cathepsin E can be involved in a variety of human diseases, such as tumors, neurodegenerative diseases and metabolic diseases.^[46,47]^ However, the role of cathepsin E in the pathogenesis of AMD needs further study. Additionally, Cathepsin G and cathepsin O were both found to significantly increase risk of in wAMD by multiple MR analysis. Cathepsin G is a serine protease primarily produced by neutrophils and can play a role in inflammation related diseases by participating in immune responses.Previous studies have found that the expression and activity of Cathepsin G in neutrophils of COVID-19 patients are related to the severity of the disease and may be significantly increased in severe patients, indicating the mechanism of action of neutrophil Cathepsin G in the thrombosis of COVID-19 patients.^[48–50]^ As for wAMD, Cathepsin G may regulate the production of cytokines and chemokines, activating and shedding cell surface receptors, and promoting apoptosis.^[14,36,37]^ For cathepsin O, it is a cysteine protease capable of catalyzing the cleavage of peptide bonds, thereby contributing to protein degradation, and it may degrade the extracellular matrix and facilitating angiogenesis to participate in the pathogenesis of wAMD. Besides, Cathepsin O may play a regulatory role in the immune microenvironment, influencing the interactions between diseased cells and immune cells, thereby contributing to the pathogenesis of wAMD.^[19,20]^ More importantly, populations may vary in their genetic and environmental characteristics, which can affect the generalizability of the results. Different subgroups within the population may have different causal relationships between the exposure and the outcome. Moreover, many diseases have complex pathophysiological mechanisms that may not be fully captured by genetic variants. Therefore, while reverse MR analysis is a valuable approach for exploring causal relationships, it needs to be carefully considered when interpreting the results. In our study, we have accounted for the specific subtype of AMD) and incorporated the most extensive publicly available dataset to enhance the accuracy of the reverse MR results.

In conclusion, the primary genetic evidence from this study reveals that high levels of cathepsin B increase the risk of AMD, particularly dAMD. This may provide a novel biochemical marker (cathepsin B) for the prediction, screening, early diagnosis, and prognosis of dAMD. Further study that pays more attention to the level of cathepsin B in the aqueous or plasma of dMAD patients. However, our study has some limitations. Firstly, the data are sourced from the INTERVAL study involving participants from European descent, which may not be generalizable to other populations. Also, the use of 2 dataset and the AMD have complex pathophysiological mechanisms that may not be fully captured by genetic variants can cause inaccurate to some extent. Secondly, although our findings have identified causal relationships between certain cathepsins and AMD, the underlying mechanisms remain unclear. Thirdly, while reverse MR analysis did not find a reverse causal relationship between cathepsin B and AMD, it still needs to be carefully considered when interpreting the results, due to the population heterogeneity, confounding factors and external validity. Finally, as with all MR analyses, our results are vulnerable to violations of the assumptions of MR and no MR result in isolation can be categorically regarded to prove causation. Understanding the specific mechanisms by which cathepsins contribute to AMD will be instrumental in developing targeted therapies for these conditions.

## Author contributions

**Conceptualization:** Jin Yang.

**Data curation:** Zhixiang Hua.

**Funding acquisition:** Jin Yang.

**Supervision:** Han Chen, Jin Yang.

**Validation:** Xiaoyan Han.

**Visualization:** Zhixiang Hua.

**Writing – original draft:** Xiaoyan Han, Zhixiang Hua.

**Writing – review & editing:** Han Chen, Jin Yang.

## References

[1] Guymer RH Campbell TG. Age-related macular degeneration. Lancet. 2023;401:1459–72. 36996856 10.1016/S0140-6736(22)02609-5

[2] Fleckenstein M Schmitz-Valckenberg S Chakravarthy U. Age-related macular degeneration: a review. JAMA. 2024;331:147–57. 38193957 10.1001/jama.2023.26074 PMC12935482

[3] Thomas CJ Mirza RG Gill MK. Age-related macular degeneration. Med Clin North Am. 2021;105:473–91. 33926642 10.1016/j.mcna.2021.01.003

[4] Cho Y-K Park D-H Jeon I-C. Medication trends for age-related macular degeneration. Int J Mol Sci . 2021;22:11837. 34769270 10.3390/ijms222111837 PMC8584051

[5] van Lookeren Campagne M LeCouter J Yaspan BL Ye W. Mechanisms of age-related macular degeneration and therapeutic opportunities. J Pathol. 2014;232:151–64. 24105633 10.1002/path.4266

[6] Girgis S Lee LR. Treatment of dry age-related macular degeneration: a review. Clin Exp Ophthalmol. 2023;51:835–52. 37737509 10.1111/ceo.14294

[7] Nashine S. Potential therapeutic candidates for age-related macular degeneration (AMD). Cells. 2021;10:2483. 34572131 10.3390/cells10092483 PMC8464988

[8] Berti PJ Storer AC. Alignment/phylogeny of the papain superfamily of cysteine proteases. J Mol Biol. 1995;246:273–83. 7869379 10.1006/jmbi.1994.0083

[9] Chwieralski CE Welte T Bühling F. Cathepsin-regulated apoptosis. Apoptosis. 2006;11:143–9. 16502253 10.1007/s10495-006-3486-y

[10] Tran AP Silver J. Cathepsins in neuronal plasticity. Neural Regen Res. 2021;16:26–35. 32788444 10.4103/1673-5374.286948 PMC7818855

[11] Rossi A Deveraux Q Turk B Sali A. Comprehensive search for cysteine cathepsins in the human genome. Biol Chem. 2004;385:363–72. 15195995 10.1515/BC.2004.040

[12] Stoka V Turk V Turk B. Lysosomal cathepsins and their regulation in aging and neurodegeneration. Ageing Res Rev. 2016;32:22–37. 27125852 10.1016/j.arr.2016.04.010

[13] Lutgens SPM Cleutjens KBJM Daemen MJAP Heeneman S. Cathepsin cysteine proteases in cardiovascular disease. FASEB J. 2007;21:3029–41. 17522380 10.1096/fj.06-7924com

[14] Burster T Macmillan H Hou T Boehm BO Mellins ED. Cathepsin G: roles in antigen presentation and beyond. Mol Immunol. 2010;47:658–65. 19910052 10.1016/j.molimm.2009.10.003 PMC4159238

[15] Turk B Turk D Turk V. Lysosomal cysteine proteases: more than scavengers. Biochim Biophys Acta. 2000;1477:98–111. 10708852 10.1016/s0167-4838(99)00263-0

[16] Joyce JA Hanahan D. Multiple roles for cysteine cathepsins in cancer. Cell Cycle. 2004;3:1516–619. 15539953 10.4161/cc.3.12.1289

[17] Song T Yao L Zhu A. Cathepsin B-activatable bioactive peptide nanocarrier for high-efficiency immunotherapy of asthma. Int J Nanomedicine. 2024;19:8059–70. 39130687 10.2147/IJN.S455633 PMC11317058

[18] Mijanović O Branković A Panin AN. Cathepsin B: a sellsword of cancer progression. Cancer Lett. 2019;449:207–14. 30796968 10.1016/j.canlet.2019.02.035 PMC6488514

[19] Im E Kazlauskas A. The role of cathepsins in ocular physiology and pathology. Exp Eye Res. 2007;84:383–8. 16893541 10.1016/j.exer.2006.05.017

[20] Bühler A Berger S Bengsch F. Cathepsin proteases promote angiogenic sprouting and laser-induced choroidal neovascularisation in mice. Exp Eye Res. 2013;115:73–8. 23800510 10.1016/j.exer.2013.06.014

[21] Alizadeh P Smit-McBride Z Oltjen SL Hjelmeland LM. Regulation of cysteine cathepsin expression by oxidative stress in the retinal pigment epithelium/choroid of the mouse. Exp Eye Res. 2006;83:679–87. 16684524 10.1016/j.exer.2006.03.009 PMC1661778

[22] Hook G Reinheckel T Ni J. Cathepsin B gene knockout improves behavioral deficits and reduces pathology in models of neurologic disorders. Pharmacol Rev. 2022;74:600–29. 35710131 10.1124/pharmrev.121.000527 PMC9553114

[23] Xue Y Cai T Shi S. Clinical and animal research findings in pycnodysostosis and gene mutations of cathepsin K from 1996 to 2011. Orphanet J Rare Dis. 2011;6:20. 21569238 10.1186/1750-1172-6-20 PMC3113317

[24] He Q Zhao MM Li MJ. Hyperglycemia induced cathepsin L maturation linked to diabetic comorbidities and COVID-19 mortality. Elife. 2024;13:1–22. 10.7554/eLife.92826 PMC11329274 39150053

[25] Emdin CA Khera AV Kathiresan S. Mendelian randomization. JAMA. 2017;318:1925–6. 29164242 10.1001/jama.2017.17219

[26] Sekula P Del Greco M F Pattaro C Köttgen A. Mendelian randomization as an approach to assess causality using observational data. J Am Soc Nephrol. 2016;27:3253–65. 27486138 10.1681/ASN.2016010098 PMC5084898

[27] Spiga F Gibson M Dawson S. Tools for assessing quality and risk of bias in Mendelian randomization studies: a systematic review. Int J Epidemiol. 2023;52:227–49. 35900265 10.1093/ije/dyac149 PMC9908059

[28] Sun BB Maranville JC Peters JE. Genomic atlas of the human plasma proteome. Nature. 2018;558:73–9. 29875488 10.1038/s41586-018-0175-2 PMC6697541

[29] Kurki MI Karjalainen J Palta P; FinnGen. FinnGen provides genetic insights from a well-phenotyped isolated population. Nature. 2023;613:508–18. 36653562 10.1038/s41586-022-05473-8 PMC9849126

[30] Bowden J Holmes MV. Meta-analysis and Mendelian randomization: a review. Res Synth Methods. 2019;10:486–96. 30861319 10.1002/jrsm.1346 PMC6973275

[31] Bowden J Davey Smith G Haycock PC. Consistent estimation in mendelian randomization with some invalid instruments using a weighted median estimator. Genet Epidemiol. 2016;40:304–14. 27061298 10.1002/gepi.21965 PMC4849733

[32] Burgess S Thompson SG. Interpreting findings from Mendelian randomization using the MR-Egger method. Eur J Epidemiol. 2017;32:377–89. 28527048 10.1007/s10654-017-0255-x PMC5506233

[33] Chlabicz M Gacko M Worowska A Lapiński R. Cathepsin E (EC 3.4.23.34) – a review. Folia Histochem Cytobiol. 2011;49:547–57. 22252749 10.5603/fhc.2011.0078

[34] Harada Y Zhang J Imari K. Cathepsin E in neutrophils contributes to the generation of neuropathic pain in experimental autoimmune encephalomyelitis. Pain. 2019;160:2050–62. 31095099 10.1097/j.pain.0000000000001596 PMC6727904

[35] Zhang X Shan P Homer R. Cathepsin E promotes pulmonary emphysema via mitochondrial fission. Am J Pathol. 2014;184:2730–41. 25239563 10.1016/j.ajpath.2014.06.017 PMC4188869

[36] Burgener SS Leborgne NGF Snipas SJ Salvesen GS Bird PI Benarafa C. Cathepsin G inhibition by Serpinb1 and serpinb6 prevents programmed necrosis in neutrophils and monocytes and reduces GSDMD-Driven Inflammation. Cell Rep. 2019;27:3646–56. 31216481 10.1016/j.celrep.2019.05.065 PMC7350907

[37] Zamolodchikova TS Tolpygo SM Svirshchevskaya EV. Cathepsin G-not only inflammation: the immune protease can regulate normal physiological processes. Front Immunol. 2020;11:411. 32194574 10.3389/fimmu.2020.00411 PMC7062962

[38] de Jong S Tang J Clark SJ. Age-related macular degeneration: a disease of extracellular complement amplification. Immunol Rev. 2023;313:279–97. 36223117 10.1111/imr.13145

[39] Flores R Carneiro A Vieira M Tenreiro S Seabra MC. Age-related macular degeneration: pathophysiology, management, and future perspectives. Ophthalmologica. 2021;244:495–511. 34130290 10.1159/000517520

[40] Shimada N Ohno-Matsui K Iseki S. Cathepsin L in bone marrow-derived cells is required for retinal and choroidal neovascularization. Am J Pathol. 2010;176:2571–80. 20304958 10.2353/ajpath.2010.091027 PMC2861121

[41] Voisin A Monville C Plancheron A Béré E Gaillard A Leveziel N. Cathepsin B pH-dependent activity is involved in lysosomal dysregulation in atrophic age-related macular degeneration. Oxid Med Cell Longev. 2019;2019:5637075. 31885803 10.1155/2019/5637075 PMC6925809

[42] Mohamed MM Sloane BF. Cysteine cathepsins: multifunctional enzymes in cancer. Nat Rev Cancer. 2006;6:764–75. 16990854 10.1038/nrc1949

[43] Cai Z Xu S Liu C. Cathepsin B in cardiovascular disease: Underlying mechanisms and therapeutic strategies. J Cell Mol Med. 2024;28:e70064. 39248527 10.1111/jcmm.70064 PMC11382359

[44] Porter K Lin Y Liton PB. Cathepsin B is up-regulated and mediates extracellular matrix degradation in trabecular meshwork cells following phagocytic challenge. PLoS One. 2013;8:e68668. 23844232 10.1371/journal.pone.0068668 PMC3700899

[45] Nettesheim A Shim MS Dixon A Raychaudhuri U Gong H Liton PB. Cathepsin B localizes in the caveolae and participates in the proteolytic cascade in trabecular meshwork cells. potential new drug target for the treatment of glaucoma. J Clin Med. 2020;10:78. 33379277 10.3390/jcm10010078 PMC7795952

[46] Yamamoto K. Cathepsin E and cathepsin D: biosynthesis, processing and subcellular location. Adv Exp Med Biol. 1995;362:223–9. 8540322 10.1007/978-1-4615-1871-6_26

[47] Kawakubo T Okamoto K Iwata J-i. Cathepsin E prevents tumor growth and metastasis by catalyzing the proteolytic release of soluble TRAIL from tumor cell surface. Cancer Res. 2007;67:10869–78. 18006832 10.1158/0008-5472.CAN-07-2048

[48] Meng J Tang H Xiao Y. Appropriate thromboprophylaxis strategy for COVID-19 patients on dosage, antiplatelet therapy, outpatient, and postdischarge prophylaxis: a meta-analysis of randomized controlled trials. Int J Surg. 2024;110:3910–22. 38549227 10.1097/JS9.0000000000001307 PMC11175823

[49] Meng J Li X Liu W. The role of vitamin D in the prevention and treatment of SARS-CoV-2 infection: a meta-analysis of randomized controlled trials. Clin Nutr. 2023;42:2198–206. 37802017 10.1016/j.clnu.2023.09.008

[50] Violi F Bartimoccia S Cangemi R. Neutrophil Cathepsin G and thrombosis in COVID-19. Circ Res. 2024;135:350–2. 38813720 10.1161/CIRCRESAHA.124.324649

